# Coefficient of Friction and Height Loss: Two Criteria Used to Determine the Mechanical Property and Stability of Regenerated Versus Natural Articular Cartilage

**DOI:** 10.3390/biomedicines10112685

**Published:** 2022-10-24

**Authors:** Markus L. Schwarz, Gregor Reisig, Barbara Schneider-Wald, Christel Weiß, Luisa Hauk, Andy Schütte

**Affiliations:** 1 Section for Experimental Orthopaedics and Trauma Surgery, Orthopaedic and Trauma Surgery Centre (OUZ), Medical Faculty Mannheim, Heidelberg University, 68167 Mannheim, Germany; 2 Department of Medical Statistics, Medical Faculty Mannheim, Heidelberg University, 68167 Mannheim, Germany

**Keywords:** coefficient of friction, articular cartilage, height loss, regenerative therapy, autologous chondrocytes, mechanical property, large animal trial, Göttingen Minipig, controls, effect size

## Abstract

Background: The coefficient of friction (CoF) serves as an indicator for the mechanical properties of natural and regenerated articular cartilage (AC). After tribological exposure, a height loss (HL) of the cartilage pair specimens can be measured. Our aim was to determine the CoF and HL of regenerated AC tissue and compare them with those of natural AC from non-operated joints and AC from joints where the regenerated tissues had been created after different treatments. Methods: In partial-thickness defects of the trochleae of the stifle joints of 60 Göttingen Minipigs, regenerated AC was created. In total, 40 animals received a Col I matrix, 20 laden with autologous chondrocytes, and 20 without. The defects of 20 animals were left empty. The healing periods were 24 and 48 weeks. A total of 10 not-operated animals, delivered the “external” control specimens. Osteochondral pins were harvested from defect and non-defect areas, the latter serving as “internal” controls. Using a pin-on-plate tribometer, we measured the CoF and the HL. Results: The CoF of the regenerated AC ranged from 0.039 to 0.069, and the HL, from 0.22 mm to 0.33 mm. The differences between the regenerated AC of the six groups and the “external” controls were significant. The comparison with the “internal” controls revealed four significant differences for the CoF and one for the HL in the operated groups. No differences were seen within the operated groups. Conclusions: The mechanical quality of the regenerated AC tissue showed inferior behavior with regard to the CoF and HL in comparison with natural AC. The comparison of regenerated AC tissue with AC from untreated joints was more promising than with AC from the treated joints.

## 1. Introduction

### 1.1. Coefficient of Friction of Articular Cartilage

Undoubtedly, the low coefficient of friction (CoF) is one of the most impressive mechanical properties of articular cartilage (AC). Regenerated AC tissue should provide a CoF comparable to that of natural and healthy AC to ensure the functionality of chondral lesions in diarthrodial joints after treatment. Thus, the determination of the CoF of regenerated cartilage could serve as a useful ex vivo tool for the assessment of strategies for treatment [1,2,3].

Many papers have been published, and several different tribological testing procedures and devices have been described where pin-on-plate apparatuses were frequently used [4,5,6,7,8,9,10,11,12,13,14,15,16,17]. 

However, where pin-on-plate setups are concerned, fewer scientific reports dealing with cartilage on cartilage systems have been published than those dealing with cartilage against alloplastic materials, mainly glass [8,10,11,13]. We believe that there is a lack of data in the literature that focuses on the evaluation of the CoF when AC and regenerated AC are tribologically examined against cartilage. 

### 1.2. Height Loss of the Cartilage Layer after Tribological Exposure

When cartilage is tested in terms of its frictional behavior, the tissue is stressed by shear forces acting parallel to the surface [12,18]. We noticed a loss of height in the pin and plate pairing specimens when they were stressed in our pin-on-plate tribometer. This phenomenon had previously been used by Katta et al. [12] as a criterion for the damage analysis of GAG-deficient cartilage in comparison with native cartilage in a long-term friction investigation [12]. Recently, we investigated the spontaneously degenerated articular cartilage and healthy cartilage of pigs [19]. Under tribological exposure, we identified divergent behavior in terms of HL, whereas the friction forces remained low and almost constant in both groups [19]. 

Thus, the determination of the HL in tribological examinations could serve as a more general criterion of mechanical stability of the assessed AC tissue. 

### 1.3. Articular Cartilage Defect Model in the Göttingen Minipig for Regenerative Treatments

Defects in the cartilage layer are frequently covered with matrices as scaffolds, either laden with autologous chondrocytes or not [20,21].

The Göttingen Minipig (GM) is a large animal model that can provide regenerated cartilage tissue for biomechanical ex vivo examinations. We recently published the study protocol and the outcomes of a large animal trial [22]. The specimens for the frictional examinations in the present study were harvested during that trial. A special feature of the study protocol was the inclusion of a group of non-treated animals as a so-called “NAT group”. Thus, natural cartilage specimens could serve as “external” controls in addition to control specimens harvested from the operated stifle joints, which we called “internal” controls [22]. 

In the present study, a focus was the usability of the CoF to characterize regenerated cartilage rather than the results achieved by the use of the Col I matrix, which has been applied to patients for a number of years [23,24]. From the scientific point of view, we were also interested in finding the best control tissue. We based our decision to include a NAT group on reports that in operated groups, cartilage adjacent to the defect could be affected by the surgical manipulations [25]. 

We present the results of the CoFs and the stability of natural and regenerated AC in defects after treatment with Col I matrices, with or without autologous chondrocytes, and spontaneously regenerated tissue when AC defects were left empty.

### 1.4. The Effect Size

We [22] recommended the calculation of the effect size (ES) according to Cohen [26] as it can help to visualize the effectiveness of a treatment by adjusting the mean values with the standard deviations [26]. Another advantage of knowing the ESs is that it allows you to compare the results of different analyses with each other as it is possible to rank the ESs [22]. In addition, power estimation can be supported by the ES as the value of the ES had a noticeable impact on the power [22] (Schwarz et al. 2019, ibid. Figure 5).

### 1.5. Focus of the Study

The aim of the study was to calculate and measure the coefficients of frictions (CoF) and the height loss (HL) of natural articular cartilage (AC) and that of regenerated AC after different treatments of partial-thickness defects in a pin-on-plate tribometer. 

The results of both criteria, the CoF and the HL, had to be analyzed in terms of significant differences compared with the control tissues originating from untreated animals and from the operated stifle joints. Similarly, we compared the results from the operated groups to detect significant differences depending on the different treatments of the defects. 

## 2. Materials and Methods

### 2.1. Animals and Treatment

The study was approved by the ethical committee of the Regierungspräsidium (Karlsruhe, Germany) with the number: AZ 35–9185.81/G-6/11.

The animal trial was described in detail by Schwarz et al. [22].

To briefly summarize: in that trial, we assessed 70 female and skeletally adult Göttingen Minipigs which we divided into seven groups of 10 animals each. In total, 10 animals were not operated on. They were identified as the NAT group and served as the “external” control group (Table 1). The articular cartilage layers of their stifle joints were left untouched until the animals were killed 24 weeks after their inclusion in the study. Briefly, eight partial defects with a diameter of 6 mm were set in the trochleae of the stifle joints of the other animals (*n* = 60), four in each joint (Supplementary Materials Figure S1). The defects of 20 animals were filled with a Col I scaffold laden with autologous chondrocytes that had been harvested from the cartilage of the defects in the setting procedure. In total, 20 animals received Col I scaffolds without cells, and the defects in the trochleae of 20 animals were left empty. Finally, 30 of the operated animals (*n* = 60) were killed after 24 weeks, and the other 30 were killed after 48 weeks (Table 1). 

The animals were operated on twice. During the first operation, the defects were set, and in the second operation, the defects were treated with scaffolds or they were left empty. The interval between the first and the second operation was 10 days in the mean [22]. The defects of the E24w and E48w groups were only debrided in the second operation because spontaneous repair tissue was found in the defects; these defects were left empty. The repair tissue was also removed in the other groups before the scaffolds were implanted using a tissue glue (Tissucol fibrin glue, Baxter, Unterschleißheim, Germany) [22] following the instructions of the manufacturer of the Col I matrix (Fa. Amedrix, Esslingen GmbH, Esslingen am Neckar, Germany). 

The scaffolds were prepared by the Fa. Amedrix according to their protocols and were delivered ready to be implanted. In the MC24w and the MC48w groups, the Col I scaffolds were laden with 2.5 × 10^4^ cells/mL [22]. 

Where the surgical procedures, the anesthesia, the welfare, and the killing of the animals are concerned, we would like to refer to our recent publication [22].

### 2.2. Specimens

For the tribological examinations in a pin-on-plate tribometer [2], osteochondral specimens had to be isolated from the operated stifle joints. They served as pins and plates. For the tribological assessment, we randomly identified one of the eight defect areas containing the regenerated tissue from one joint of each animal. The osteochondral pin with a diameter of 5 mm was harvested from that defect area as described by Schwarz et al. [27] so that there was only regenerated tissue on top of the pin.

The tibia plateaus served as plates, and both the pins from the defect area and the non-defect area (internal control) were randomly matched to the medial or lateral site of the plateau. A custom-made square punch was used to cut out osteochondral plates with a dimension of 20 mm × 20 mm, either out of the medial or the lateral tibia plateau [2].

Using custom-made tools, the handling and preparation of the specimens were performed very carefully, so that the assessed surface areas of the cartilage were not touched [22]. The specimens were stored at −20 °C in PBS [28,29].

For the tribological examinations, pins and plates were thawed according to the protocol we had developed and validated previously with comparable osteochondral specimens from the stifle joints of pigs from the slaughterhouse. The thawing process took place in a water bath at 25 °C immediately before the tribological examinations were performed. The thawing time for the pins was 20 min and 30 min for the plates, due to their different masses. After thawing, the specimens were again stored in PBS. We waited a minimum of 10 min after thawing before we started the examinations. That allowed enough time for the specimens to acclimatize to the temperature of the room where the examinations were performed.

After we had placed the pin and plate in the tribometer, we tried to identify the flattest area of the surfaces of the plates where the trajectories of the pins were going to run. The adjustment was performed manually by moving the plate in the Y-direction. By doing so, we tried to avoid a possible impact of the unevenness of the surface of the cartilage plate on the results, as that could act as a confounder [2].

The specimens for the tribological examinations were blinded. For that purpose, the tubes containing the pins or plates were numbered in ascending order and the specimens were randomly placed into the tubes by someone other than the examiner (AS). Thus, the examiner never knew what kind of specimen he was handling at any given time. At the end of the tribological examinations, the specimens that had to be excluded were identified, CoF and HL were calculated (see below), and finally, the specimens were decoded.

### 2.3. Examinations in the Tribometer

We used the tribometer recently described by [2].

To allow an unhindered vertical displacement of the pin, the device was equipped with a lever arm construction that worked frictionlessly. The pin was pressed onto the surface of the plate by dead weights, producing a pressure of approximately 0.75 MPa between the pin and the plate by loading with 14.9 N [9,12]. The plate was moved by an X–Y table that was driven by linear actors [2,3]. The direction of the reciprocating trajectory of the pin was set in the sagittal direction of the joint, leading to a movement of the pin from ventral to dorsal and back to ventral corresponding to the anatomical situation. This trajectory was aligned along the X-direction of the coordinate system of the tribometer. The acceleration and deceleration were 50 m/s^2^. Thus, we were able to achieve a plateau value of 4 mm/s [30] between the acceleration and deceleration phases in the ramp-shaped loading diagram. The stroke length of the trajectory was 13 mm, and the calculations of the CoF were performed over a distance of 3 mm in the middle of the stroke distance. Thus, the CoF was determined in a dynamic-friction setup where, during each cycle, the plate was unloaded twice for a short period of time [4].

The tribometer was equipped with a three-axis force sensor (K3D120 50N/50N/100N, ME-Meßsysteme, Henningsdorf, Germany) with an amplifier (GSV1-H, ME-Meßsysteme, Henningsdorf, Germany) that contains a second-order low-pass filter of 250 Hz. 

We stressed the pair specimens consisting of pin and plate over the period of 1 h, which corresponded to 554 cycles. 

To measure the displacements of the pin in the vertical direction we used a laser distance sensor (ILD optoNCDT 2300-20, MicroEpsilon Messtechnik GmbH & Co. KG, Ortenburg, Germany). 

The rate of data acquisition was set at 1 kHz; the data of force and position were stored synchronously in LabVIEW (V11.0.1, National Instruments, Austin, TX, USA) using a custom-made program by National Instruments (Austin, TX, USA) [2].

### 2.4. Calculation of the CoF

The CoF was calculated as described by Schütte et al. [2]. The key component of the calculation was to take the unevenness of the articular cartilage surfaces of the tibia plateaus into account because those surfaces are naturally uneven. Thus, the different slope angles of the surface had to be included for every measurement at each point in time. The slope angle was calculated using the measurements of the distance sensor in a Z-direction and applying a two-dimensional regression analysis [2,30]. Thus, we were able to calculate the forces acting parallel to the surface as well as the forces acting perpendicular to the surface [2]. The force acting parallel to the surface represented the friction force and the force acting rectangular to the surface represented the normal force. The CoF was correctly calculated by establishing the ratio between friction force and normal force, thus reflecting this particular situation [2,31,32]. The CoF was calculated for both directions, taking the reciprocating trajectory of the pin-on-plate into account. The means of the CoF of both directions were averaged according to the recommendations of Schütte et al. [2].

### 2.5. Calculation of the HL (Height Loss)

The HL was calculated in [mm] over a distance of 10 µm in the middle of the stroke distance of each cycle. In order to determine the HL, we measured the difference between the starting point, set as a reference point, and the height for each cycle when the pin had moved over the 3 mm distance in the middle of the stroke distance. The mean of both directions was used in each cycle.

### 2.6. Exclusion Criteria of Data

Due to complications during the second operation when the defects were treated, some stifle joints, referred to as “dropouts”, could not be included in the study [22].

The tribological examinations were closely observed and documented by taking pictures. Any possible suspicious events were recorded. 

The data of a tribological examination were excluded, when one of the following problems was noticed:

- artificial damage to the cartilage at the pin or within the friction distance on the plate was macroscopically identified
- a pair specimen was stressed (loaded) before the experiment had started, so it moved through some cycles with the wrong test parameters
- the pin or plate wobbled during the friction experiment due to insufficient fixation
- the data records of the experiment showed undefined values because the position and movement direction data did not match
- if we suspected that the sample holder of the pin might have touched the cartilage surface of the plate before or during the experiment
- the testing device did not work correctly

Apparent degenerative changes of the cartilage layers of the plate were no exclusion criteria.

### 2.7. Statistics 

A sample size calculation was performed before the start of the animal study (for details, see Schwarz et al. [22]). Briefly, 10 animals had to be included in each group. 

The ES for the different results regarding the CoF and the HL of the NAT group and the operated groups was calculated according to Cohen [26].

The system identified the slope angles of the uneven areas of the plate as negative (−) or positive (+). For the description of the ranges of the values of the angles, we transformed all values into (+) so that the lowest angle was not lower than 0°. Quantitative variables are presented by mean ± standard deviation.

We used box and whiskers plots for the descriptive statistics, performed with the Origin 8.6.0G software (OriginLab Corporation, Northampton, MA, USA). The box covers the interquartile interval; the line inside the box is the median; the mean value is shown by the square; the whiskers indicate the minimum and the maximum values.

We used the Wilcoxon Rank Sum test for paired and for unpaired samples as appropriate. 

We performed the statistical comparisons regarding the CoF and the HL between the control group and the treated groups for each point in time. In the treated groups, we performed pairwise comparisons between the E24w group and E48w, and between the M24w with the MC24 group. Furthermore, the E48w group was compared with the M48w and the MC48w groups. The M24w group was also compared with the M48w and the MC24w groups. In addition, the M48w group was compared with the MC48w group and the MC24w group with the MC48w group.

The level of significance was set at 0.05.

## 3. Results

### 3.1. Complications and Welfare of the Animals

We would like to refer to our recently published report where the complications of the study and the welfare of the animals were described in detail [22]. To briefly recapitulate, we lost seven animals in the study that were replaced. Two stifle joints from two different animals and two different groups (E24w and MC48w) were operated on only once due to infections, thus leading to the “dropouts” in the present study.

### 3.2. The Specimens

In total, 140 pin specimens were assessed. Nine examinations with specimens from the defect area and five with specimens from the non-defect area were excluded from the analyses, due to the described exclusion criteria. We ended up with 90% valid values in total.

### 3.3. Quality of the Cartilage Pin and Plate; Slope Angles of the Uneven Plate

We assessed the unevenness of the plates in the measuring distance by determining the slope angles. In all assessed examinations, we identified slope angles lower than 15° with a maximum of 14.99° (minimum: 1.3 E-8°), a median of 2.62°, a mean of 3.21°, and a standard deviation of ±2.52°. 

**Figure 1:**
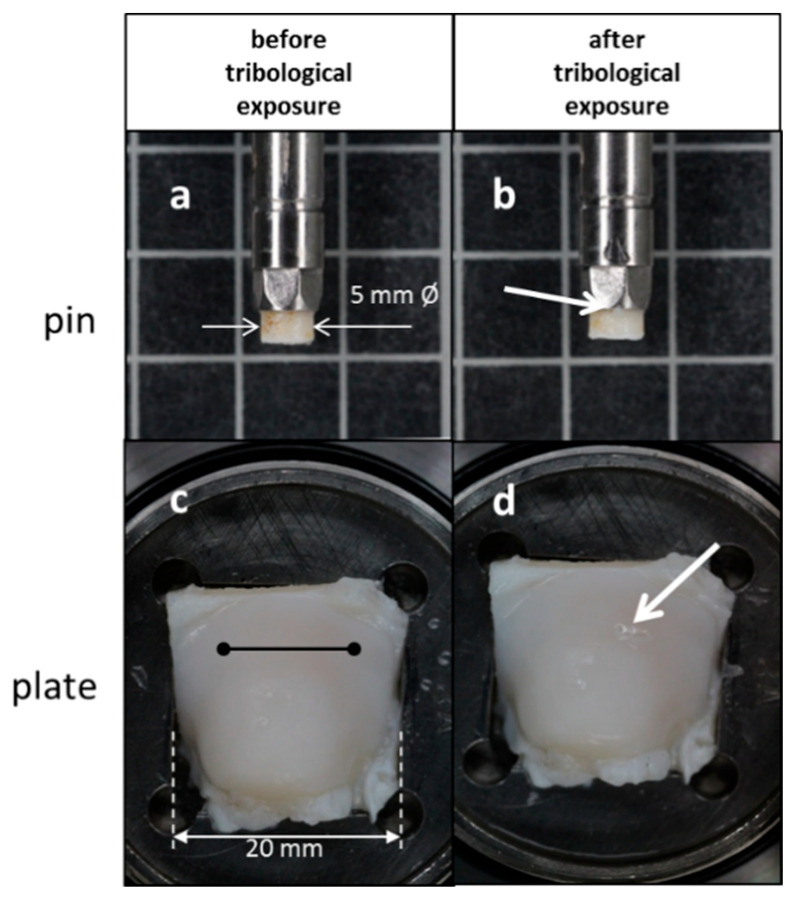
Pin and plate pair specimen from the E48w group before and after tribological exposure. A CoF of 0.0287 was calculated in the mean. (**a**) The pin is fixed in the pin holder. A thin layer of spontaneously regenerated tissue is visible on top of the pin after the healing period of nearly 1 year. (**b**) A small shred of a mucous consistency that detached itself from the layer stuck to the rim of the pin holder (arrow). (**c**) Top view on the plate originating from the lateral tibia plateau. The trajectory of the pin is indicated by the black line. The analysis of the uneven surface of the section where the pin moved revealed slope angles ranging from 0.00° to 7.65° with a median of 2.16°. (**d**) After the tribological exposure, some irregularities of the surface were noticeable (arrow). The height loss (HL) was determined with 0.35 mm in the mean in the middle of the trajectory after 1 h of reciprocating shear stress. Note the somewhat different magnifications of the figures.

Figure 1 gives a qualitative impression of a pin and plate pairing of the E48w group before and after the tribological examination.

### 3.4. Coefficient of Friction

#### 3.4.1. Defect areas

**Figure 2:**
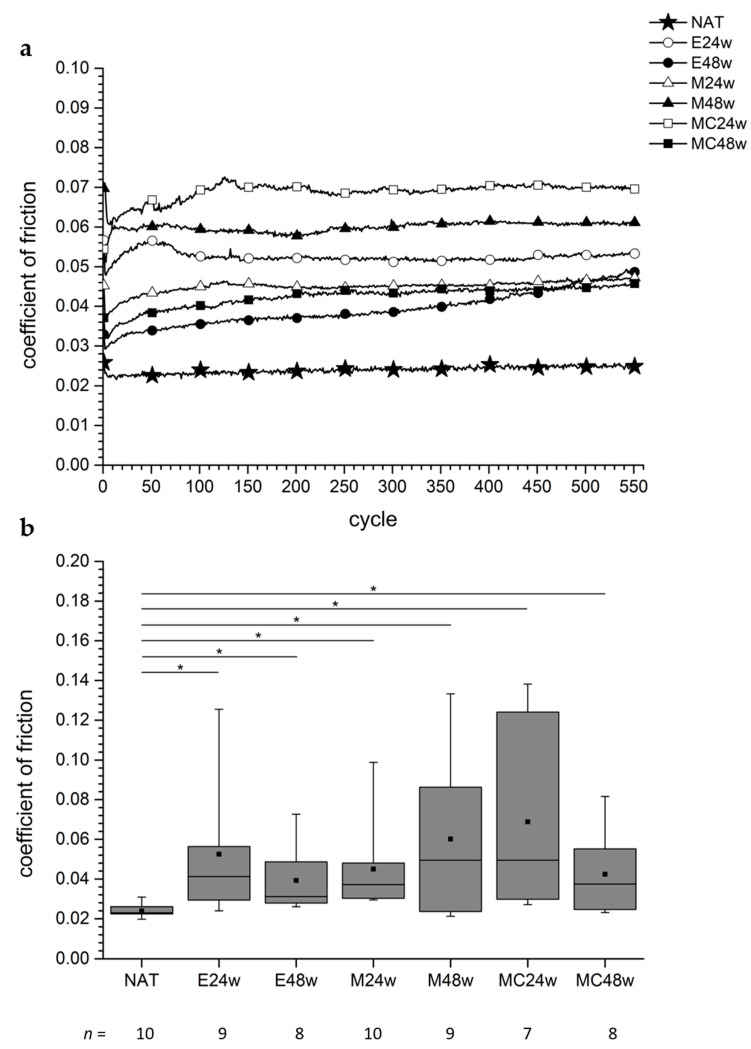
The figure shows the CoFs of the specimens from the defect areas. (**a**) The CoF of the specimens from the NAT group reveals a low and constant value during the observation time of the examinations of approximately 1 h (554 cycles). The regenerated tissues deliver higher CoFs with a mainly asymptotic progression. For the sample size per group, see figure (**b**). (**b**) All CoFs of the regenerated tissue from the operated groups are significantly higher than those of the AC from the NAT group. The * shows the significance between the two groups.

The behavior of the CoFs of the natural cartilage (NAT) was quite constant during the observation time of approx. 1 h (Figure 2). The CoFs of the defect areas of the operated groups (E24w, M24w, M48w, MC24w, and MC48w) showed a slight increase with an asymptotic behavior, whereas the CoFs of the regenerated cartilage of the E48w group increased after half the time of the examination had elapsed (Figure 2a). 

The CoF of the regenerated cartilage in the defect areas ranged from 0.039 ± 0.017 (E48w) to 0.069 ± 0.045 (MC24w). The CoF in the defect areas of the NAT group was 0.024 ± 0.003. 

The NAT group revealed the lowest CoF, and the CoFs of all operated groups were significantly higher than those of the NAT group (NAT vs. E24w: *p* = 0.0015, NAT vs. E48w: *p* = 0.0009, NAT vs. M24w: *p* < 0.0001, NAT vs. M48w: *p* = 0.0350, NAT vs. MC24w: *p* = 0.0004, NAT vs. MC48w: *p* = 0.0155) (Figure 2b). 

The pairwise comparisons regarding the CoFs of the operated groups revealed no significant differences in all assessed groups. The *p*-values ranged from *p* = 0.281 to *p* = 1.0.

#### 3.4.2. Non-Defect Areas

Over the entire observation time, the values of the CoF of the non-defect areas were quite similar for all groups and showed a constant behavior (Supplementary Materials Figure S2a). 

In the non-defect areas, the CoF was 0.027 ± 0.007 in the NAT group, and in the operated groups, the CoF ranged from 0.025 ± 0.002 (E24w) to 0.027 ± 0.006 (MC48w). There were no significant differences between the results of the NAT group and those of the operated groups (Supplementary Materials Figure S2b). The *p*-values ranged from *p* = 0.6607 to *p* = 1.0. 

The CoFs of the operated groups revealed no significant difference within the assessed groups, with *p*-values ranging from 0.2475 to 0.9314.

#### 3.4.3. Comparison between Defect and Non-Defect Areas in the Groups

The comparison between the CoFs of the tissue from the defect area and the tissue from the non-defect area in the NAT group showed no significant difference (*p* = 0.4258). In the NAT group, the CoF of the tissue from the non-defect area was slightly higher (0.0266 ± 0.0068) than the CoF of the tissue from the defect area with 0.024 ± 0.0031. 

In all operated groups, the CoFs of the tissue of the non-defect areas were lower than those of the defect areas.

The comparison between the CoF of the regenerated cartilage tissue from the defect areas in the operated groups and the CoF of cartilage tissue from the non-defect areas revealed significant differences in four cases (E24w: *p* = 0.0273, E48w: *p* = 0.0156, M24w: *p* = 0.002 and MC24w: *p* = 0.0313) but not in the M48w group (*p* = 0.0742) and the MC48w group (*p* = 0.0547).

### 3.5. Height Loss

#### 3.5.1. Defect Areas

**Figure 3:**
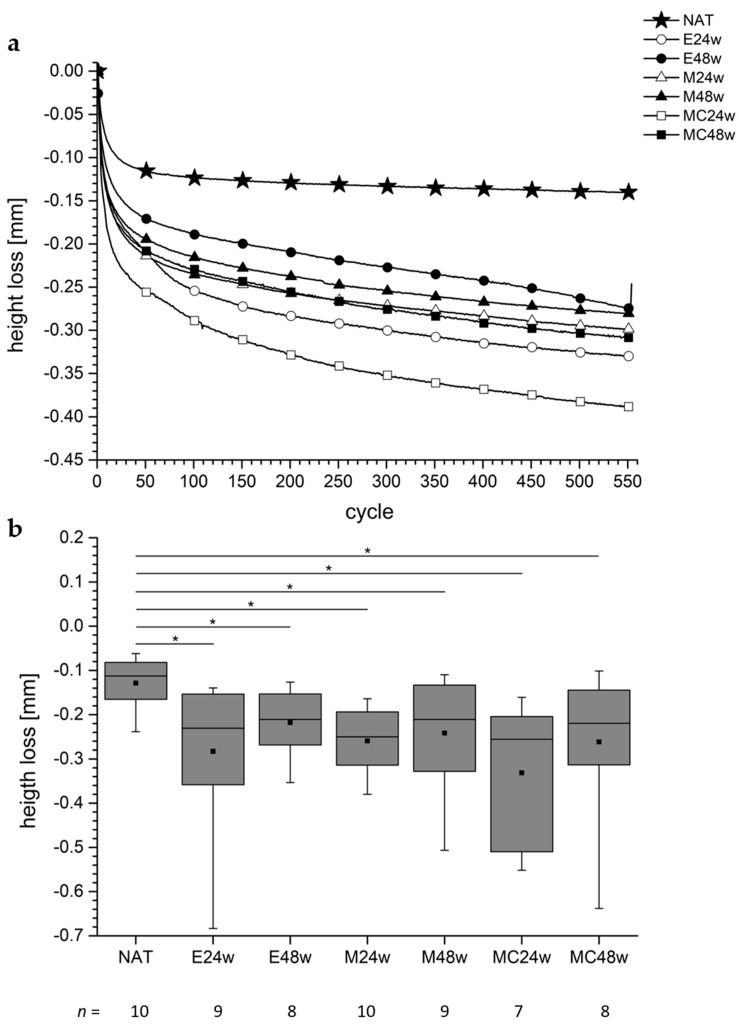
The figure shows the HLs of the specimens from the defect areas. (**a**) The trends of the HLs of the specimens from the defect areas over the duration of the examinations of approximately 1 h (554 cycles). The regenerated tissues reveal a higher and increasing HL over the time of observation. The specimens from the NAT group show a lower and not substantial further increasing HL after approximately 50 cycles. For the sample size per group, see figure (**b**). (**b**) All HLs of the regenerated tissue from the operated groups are significantly higher than those of the AC from the NAT group. The * shows the significance between the two groups.

There was a pronounced HL within the duration of the first 50 cycles (Figure 3a). The NAT group showed the lowest HL of the tissue taken from the defect areas; the curve had an asymptotic character but the curves of the treated groups seemed to continue to descend. 

There was an HL of the tissue from the defect area of the NAT group of 0.13 mm ± 0.06 mm, and in the operated groups, the HL ranged from 0.22 mm ± 0.08 (E48w) mm to 0.33 mm ± 0.16 mm (MC24w).

The HL of the tissue from the defect areas of the NAT group and the tissue from the defect areas of the operated groups differed significantly (Figure 3b). (NAT vs. E24w: *p* = 0.0133, NAT vs. E48w: *p* = 0.0266, NAT vs. M24w: *p* = 0.0011, NAT vs. M48w: *p* = 0.0220, NAT vs. MC24w: *p* = 0.0012, NAT vs. MC48w: *p* = 0.0205).

The comparison of the tissues from the defect areas of the operated groups showed no significant differences (*p*-values ranged from *p* = 0.3357 to *p* = 0.9626).

#### 3.5.2. Non-Defect Areas

In all groups, there was a pronounced HL of the tissue from the non-defect areas during the first 50 cycles, followed by an asymptotic behavior (Figure S3a). During the period of observation, the tissue of the NAT group lost slightly more height than that of the MC48w group (Figure S3a). 

The tissue from the non-defect area of the NAT group showed an HL of 0.15 mm ± 0.06 mm, and in the operated groups, the HL ranged from 0.14 mm ± 0.05 mm (MC48w) to 0.21 mm ± 0.11 mm (E48w). 

There was no significant difference between the HL of the tissue taken from the non-defect areas of the operated groups and that taken from the NAT group (*p*-values ranged from 0.1903 to 0.9682) (Figure S3b).

The comparison of the HLs of the regenerated cartilage tissue of the operated groups showed no significant differences (*p*-values ranged from *p* = 0.1135 to *p* = 0.9314).

#### 3.5.3. Comparison between Defect and Non-Defect Areas in the Groups

In the NAT group, there was no significant difference (*p* = 0.5703) in the HL between the tissue taken from the defect areas (0.13 mm ± 0.06 mm) and that taken from the non-defect areas (0.15 mm ± 0.06 mm). Only in the M24w group was there a significant (*p* = 0.0137) difference in the HL between the tissue taken from the defect areas and that taken from the non-defect areas, with 0.18 mm ± 0.06 mm for the latter and 0.26 mm ± 0.07 mm for the former. In the other groups, no significant differences were seen (*p*-values ranged from 0.0625 to 0.4688).

### 3.6. Effect Sizes (ES)

We determined the ESs for both the CoF and the HL. 

**Figure 4:**
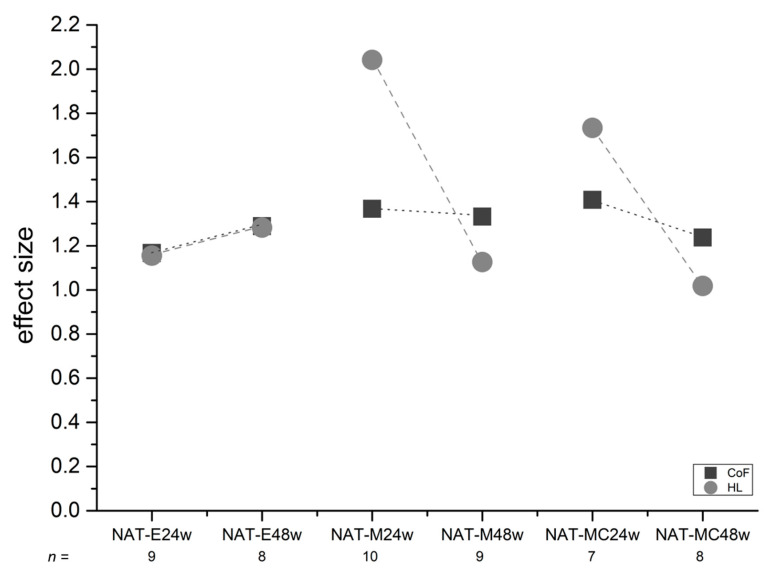
The effect sizes (ESs) of the CoF and HL were calculated for the comparisons between the NAT group and each of the operated groups.

The ES of CoF ranged from 1.17 to 1.41 (mean: 1.30; standard deviation: ±0.08 (Figure 4)), with the highest value in the comparison between the NAT group and the MC24w group and the lowest value in the comparison between the NAT and the E24w group. 

The ES of HL ranged from 1.02 to 2.04 (mean: 1.39; standard deviation: ±0.37 (Figure 4)), with the highest value in the comparison between the NAT group and the M24w group, and the lowest value in the comparison between the NAT with the MC48w group. 

Thus, all ESs were >0.8 leading to the ranking “large effect” according to Cohen [26]. Regarding the sample size calculation procedure as described in our former study [22], the power estimation was in a range from approx. 60% to approx. 80% and higher Schwarz et al. 2019 [22] (Schwarz 2019, ibid. Figure 5).

An ES > 0.8 is ranked as large [26]. Thus, important ESs were detected in all cases. Regarding the two different healing periods (24 weeks and 48 weeks), the empty groups (E24w and E48w) revealed a mild deterioration in terms of the CoF and HL. The operated groups showed improvement after 48 weeks. 

## 4. Discussion

The aim of the study was to analyze the CoF and the HL after tribological exposure of regenerated articular cartilage after different treatments. Another aspect was to find out if it was possible to use internal controls taken from the same trochlea facet where the tissue regeneration had taken place, or if it was necessary to use natural cartilage as external control specimens from untreated animals for tribological examinations for better differentiation. The comparison of the statistical outcomes of the applied types of controls might provide an answer to that question. 

### 4.1. CoF, HL, and Controls

The low CoF of articular cartilage is a frequently assessed quality of this tissue and could serve as an appropriate parameter for the validation of treatment for articular cartilage regeneration regimes [2,3,11,14,16]. Link et al. [14] interpreted the current situation of published data as “under-characterized” in terms of a “replication of tribological properties” of tissue-engineered constructs. 

The determination of the CoF of articular cartilage has been described in several publications with the aim to understand its mechanical property in particular and to determine the impact of pathological changes of the tissue [12,33,34,35]. The aim of regenerative medicine is the restoration of lost or damaged tissue. Thus, in the case of AC, the regenerated tissue is expected to have quite similar friction properties to healthy AC [11,36]. However, we received the impression that there is a lack of data in the literature regarding tribological examinations of regenerated AC tissue that was tribologically tested with AC as a counterpart. AC and regenerated AC were mostly tested against alloplastic materials such as glass [8,10,11,13]. By testing AC against AC, we hoped to get more specific CoF values, as the CoF curves in such examinations revealed a constant and lower CoF over an observation time of 1 h [4,37], 8 h [17], or even 15 h [38]. For the tribological examination of the regenerated AC that we produced in the Minipig Model defect model [22], we developed and validated a custom-made device for the friction analysis of AC and AC regenerates against AC [2].

We detected a measurable HL of the pair specimens in the tribometer via the integrated distance sensor for the Z-direction used for the calculation of the unevenness of the plate surface [2,3,19]. 

We decided to take the HL as a surrogate criterion for the biomechanical qualities of AC and regenerated AC even if it was not possible to distinguish between the different mechanical properties like moduli or the hydraulic permeability in particular [39]. In addition, we needed to take into account the loss of tissue through wear because we noticed that tissue on the surface of the cartilage layers was abraded during tribological exposure [19,38,40] (Figure 1). 

Thus, an analysis of the mechanical quality of the regenerated cartilage could be attempted using the results gathered during tribological exposure when shear stress was applied [12]. 

From the biological point of view, it was not clear if the cartilage adjacent to the defect areas could serve as internal controls or if it could have been altered by the surgical manipulations [25] and hence would not be suitable. 

With this context in mind, we needed to decide what counterpart of the pin to take for the examinations in the tribometer. We had the choice between the communicating facets of the patella because the pins were taken from the facets of the trochleae or the tibia plateaus of the same joint. According to Schinhan et al. [41], one has to consider degenerative reactions at the joint surface corresponding to the surface where the defects were set [41]. This phenomenon is addressed as “kissing lesions” in joint surgery [42]. The fact that the facets of the patella are small was another disadvantage because it was not possible to harvest osteochondral plugs with a dimension of 20 mm × 20 mm. In addition, the curvature of the surface of the facets of the patella tend to show higher slope angles than the curvature of the tibia plateaus [2]. Thus, we decided to perform the CoF measurements with pair specimens consisting of a pin and a plate from a part of the tibia plateau, medial or lateral. 

### 4.2. Animal Model

The regeneration of articular cartilage was performed in a large animal model with GMs [22]. Cone et al. [43] reported an increasing use of pigs in musculoskeletal research relating to areas like cartilage tissue, tissue engineering and regenerative procedures, biomechanics, specific joints, etc. in recent years [43]. The comparability of articular cartilage research of this large animal model with the musculoskeletal system of humans has been previously described and discussed in several publications [44,45,46]. Similar to human articular cartilage, spontaneous degenerations of the articular cartilage have also been observed in the stifle joints of pigs [45,47,48]. 

### 4.3. Lost Values 

The values that had to be excluded were identified before the blinded specimens were decoded. Unfortunately, we lost 10% of the values (14/140). However, as five of those values resulted from the non-defect areas serving as internal controls, the loss of values amounted to nearly 13% (9/70) for the defect areas. The reasons for the exclusion were mostly technical problems like the fixation of the pin or plate in the tribometer. Some were excluded because of complications during surgery [22].

### 4.4. Sample Size

According to the sample size calculation, we started the animal study with 10 animals in each group (Table 1) as recently reported by our working group [22]. The MC24w group showed the highest loss, namely three values. In the other groups, no more than two values were lost altogether. However, significances were detected between the operated groups and the NAT group, and we think that the lack of significances within the operated groups is mainly caused by the wide spread of the data rather than by reduced sample size. However, 10 animals in each group was obviously a realistic number for detecting significance. 

### 4.5. Lubricant PBS 

The results of a tribological examination can be affected by different parameters. One important factor is the choice of the lubricant [49]. Looking at different aspects, we believed that PBS was the best choice for our study. As PBS was prepared in the laboratory under reproducible and reliable conditions, we could assume that the quality of the lubricant was consistent. However, studies have shown that PBS could alter the tissue or affect the CoF in examinations. This would not be the case with synovial fluid (SF) [50,51]. However, the composition of SF is variable and can depend, for example, on the grade of osteoarthritic (OA) degenerations, as Kosinska et al. have shown [52]. They reported that hyaluronic acid, lubricin, and phospholipids play a crucial role in the SF of joints, depending on the condition of the joint [52]. We also needed to keep in mind that pigs could suffer from OA [22,45,47,48]. The use of SF could actually hide a different tribological effect like the CoF of regenerated cartilage tissue because the use of SF could induce a decrease in CoF. Caligaris et al. [7] reported that the use of SF decreased the friction coefficient of human OA-altered cartilage; this was not the case when PBS was used as a lubricant [7].

According to the report by Wong et al. [18], PBS could also help reveal weaknesses in the assessed tissue because it would lead to more rigorous examination conditions with regard to the applied shear stress. Thus, the determination of HL as a surrogate criterion for the stability of natural or regenerated cartilage could help to better assess the tissue when using PBS as a lubricant. 

It was not the aim of the present study to actually determine the “true” CoF value of the cartilage but to find out if there were differences in CoF between varying conditions of cartilage tissues. Furthermore, the examination protocol with regard to the CoF needs to be practicable and reliable, and the results need to be comparable to be helpful in future studies.

### 4.6. CoF 

The CoF is considered to be an important biomechanical parameter of articular cartilage necessary for the smooth functioning of natural and regenerated cartilage tissue.

#### 4.6.1. CoF in Pin-on-Plate Tribometers

Pin-on-plate tribometers are frequently used for the calculation of the CoF even when cartilage is stressed against cartilage [1,4,9,38,53,54]. In the present study, we used a tribometer that had been specifically developed for the examinations of cartilage or regenerated cartilage against cartilage. It addresses the natural unevenness of the plate, a fact that had to be considered for the calculation of the CoF [2]. Pin-on-plate tribometers cannot perfectly reflect the physiological situation of a joint as it does not also imitate the rolling motion in the joint. However, the use of the pin-on-plate tribometer specifically allowed the examination of regenerated cartilage tissue because it was possible to assess the isolated tissue in form of the layer on top of the osteochondral pin [27].

#### 4.6.2. CoF in Literature 

In the present study, we measured a CoF of natural cartilage of 0.024 in the mean. This was similar to the CoF of µeff = 0.018 to µeff = 0.044 reported in the literature by Caligaris and Ateshian [6]. Northwood and Fisher [17] reported a friction coefficient of 0.04 to 0.05. The results were gained in comparable experimental setups, using pin-on-plate tribometers [6,17]. Kanca et al. [38] found a CoF of 0.03 in a biaxially working pin-on-plate device [38]. Arakaki et al. [1] investigated the friction of a double-network hydrogel construct against cartilage and found a CoF of 0.029 in comparison to that of 0.188 when they tested cartilage against cartilage from the knee joints of rabbits [1]. 

Basalo et al. [55] stressed bovine cartilage plugs, 4.78 mm in diameter, with a pressure of 0.5 MPa, and found an increasing friction coefficient when glycosaminoglycans were removed with chondroitinase ABC. The loading conditions were constant over the observation time of about 41 min [55]. However, the results of Basalo et al. [55] are not completely comparable with the results of the present study because they removed the deep zone of the cartilage. In their studies, the friction coefficient increased from 0.037 to 0.12 for the untreated cartilage and from 0.0053 to 0.19 for the treated cartilage [55]. In the present study, after a period of time, the CoF remained almost constant in most cases (Figure 2a). We think that the friction coefficient increased with Basalo et al. [55] as they used glass as the tribological counterpart for the cartilage. These testing conditions deliver increasing friction coefficients, as has also been observed in other studies [4,17,37,38]. 

Griffin et al. report CoFs ranging from 0.42 to 0.52 of full-thickness cylindrical plugs 3 mm in diameter, including the controls from the non-operated contralateral knee joints of the horses [11]. They investigated the biomechanical properties of “matrix-induced autologous chondrocyte grafts” after a healing time of 53 weeks. The comparison of the CoFs found in their study with the CoFs found in the present study shows that the CoFs obtained with glass as a friction counterpart delivered higher values than those that we identified [11]. They found some significant differences for the aggregate modulus, the hydraulic permeability, and the shear modulus, showing that the regenerates differed from the controls in terms of some of the biomechanical properties but not for the CoF [11]. In contrast, in the present study, significant differences were seen between the NAT and the operated groups (Figure 2b). The assumption that higher CoFs are found when cartilage was moved against glass or steel has been confirmed by several studies [14], especially when both procedures were performed [17,38,56].

Thus, in the present study, we used a more authentic situation when cartilage was moved against cartilage [2], leading to constant and lower CoFs.

The curve progression of the CoF in the NAT group in this study, shown in Figure 2a, resembles curve progressions shown in the literature when cartilage was moved against cartilage [17,38], in particular under dynamic friction conditions [4]. The CoF is quite constant over the period of observation, namely, 0.024 in the mean with a standard deviation of 12.5% (with ±0.003). This differed from the results of the regenerated tissue. 

It is remarkable that none of the assessed operated groups showed the same low CoF as the natural cartilage of the NAT group. Their CoFs were all significantly higher (Figure 2b). Unfortunately, the information on the CoFs of regenerated cartilage tissue in the literature, in particular on spontaneously regenerated cartilage ex vivo, is rare. Thus, the comparison of the results of regenerated cartilage in this study with that of other studies is limited. 

#### 4.6.3. CoF Regarding Internal and External Controls

When planning an animal study to analyze the effectiveness of a treatment, valid controls must be identified. When we planned the present study, we did not know if the CoF of an articular cartilage surface would be affected by manipulations in the same knee joint where the treatment was applied, similar to what Strauss et al. [25] described for other mechanical properties of articular cartilage [25]. We decided to create four defects in each trochlea of the stifle joints of GMs [22], and we saw that osteochondral pins (Ø 5 mm) could be harvested next to the defect areas as controls, which we called “internal controls” (Figure S1). However, we were not sure if the frictional quality of the adjacent cartilage could have suffered due to the surgical treatment. One has to take into account that we operated each joint twice and the second operation could take up to 3 h [22]. 

We could not confirm our hypothesis because we did not find any significant differences between the cartilage tissue from the non-defect areas of the NAT group or the cartilage tissue of the operated groups. However, the comparison of the regenerated cartilage of the treated groups with the natural cartilage of the NAT group revealed significant differences in all six cases (Figure 2b). 

We only found four significant differences with regard to the CoF when comparing the regenerated cartilage tissue with the internal controls of the operated groups (E24w, E48w, M24w, and MC24w). Thus, the use of natural cartilage of an untouched joint as external control seems to be preferable because regenerated articular cartilage tissue should have tribological properties very similar to those of natural AC tissue.

#### 4.6.4. Validity of the CoF Calculation

The validity of the measured and calculated values of the CoF depends on several issues. The selection of the assessed specimens was performed randomly, and so was the choice of either the medial or the lateral tibia plateau as a counterpart for the pins (see above). The preparation of the specimens was carefully executed using custom-made tools, thus ensuring that the surfaces of the cartilage or the regenerated tissue were not touched [22]. Only one person (AS) performed all examinations. For the validation of the results of the CoF, the impact of an uneven surface was addressed in the measurements and calculations in the tribological system. In this context, we can state that in the present study, the CoF measurements and calculations were obtained in the confidence range of a slope angle up to 15° because the highest slope angle was even lower (14.99°). The median of the slope angles was 2.62°, and the mean was 3.21° with a standard deviation of ±2.52°. We believe that the calculated values are highly reliable, and the low spread of data of the external and internal controls supports this assumption (Figure 2b and Figure S2b).

### 4.7. HL 

The determination of the HL of the articular pair specimens is a procedure that could serve to judge the stability of the natural and degenerated [19] and perhaps also of regenerated articular cartilage tissue.

#### 4.7.1. HL after Applied Shear Stress

When articular cartilage slides against articular cartilage, shear forces and shear stress occur, and, as a consequence, energy is dissipated [18,57]. Shear kinematics in such a setup have been previously explained by Wong et al. and Katta et al. [12,18]. In the present study, we found that the shear stress applied to the tissues led to a measurable HL in all assessed groups, and this led us to assume that in addition to the loss of water [39], energy dissipation occurred because boundary friction takes place when solid partners are in contact [57]. The HL was least noticeable in the NAT group with 0.13 mm in the mean over the examination time, and the asymptotic curve revealed a stable situation after approx. 150 cycles (Figure 3a). The worst HL was seen in the MC24 group, and the most interesting group was the E48w group. It revealed a curve with an increase in HL after approx. 400 cycles. Cartilage tissue obviously matured spontaneously in the E48w group, and the characteristics of the HL curve could reveal a kind of fatigue process of that tissue (Figure 3a). It was only in this group that the CoF curve ascended (Figure 2a). It seems that the HL correlated uniformly with the CoF in this group, starting approximately at cycle 400 (Figure 2a). This observation could support the hypothesis that the higher the CoF the higher the energy dissipation [57]. However, the curves of the CoF of the other five operated groups in this study revealed an asymptotic shape while the HL curves continued to decrease, reflecting an increase in the HL (Figure 2a and Figure 3a). Looking at our histological outcomes, we would like to refer to a recent publication of our working group (see Figure 7 in Schwarz et al. [22]) where the E48w group showed the worst histological outcome in the scoring according to O’Driscoll et al. [58]. Thus, it is likely that the treated groups could benefit from the implantation of matrices after 1 year as opposed to the non-treated groups (Figure 4). 

The phenomenon that there is a pronounced HL in all groups during the first 50 cycles may be explained by the setting processes of the pair specimens. As we started measuring when the pair specimens were pressed together with 0.75 MPa, we think that setting movements of the osteochondral pins and plates in their holding devices are not likely after the application of pressure. However, because of the unevenness of the plate, setting processes in the contact areas of the pins and plates cannot be excluded. 

We assume that the measured HL in the present study depended largely on the material property of the pin with the regenerated AC tissue on top. The pin was always under load, and thus it had no chance for recovery in terms of rehydration. On the other hand, the plate was exposed to a migrating contact condition [59], and the AC tissue that the pin moved across in a reciprocating manner could recover. Bell et al. [4] report that the AC layer of the plate could be “replenished” if the experiments were performed under dynamic friction conditions as opposed to static conditions. In the present setup, we calculated the CoF only under dynamic conditions in the middle of the stroke distance so that static impacts at the turning points could be excluded [2,4]. Thus, the pin was likely more highly stressed in the present setup than the AC tissue of the plate. However, it is not clear if the entire surface area of the pin was continuously under stress when it moved over the uneven surface of the plate. Therefore, parts of the surface area of the pin could have been under load or not, depending on the contact conditions and the direction of movement. It is possible that the pin as well could benefit from tribological rehydration arising under these circumstances, thus leading to a lower HL [35]. 

Nevertheless, our findings regarding the measured HL led us to believe that the applied protocol could serve as a testing procedure to determine the resilience of tissue because the ‘weaker’ the tissue, the higher the HL.

#### 4.7.2. HL in Literature 

The HL was the topic of a study published by Katta et al. [12] where they analyzed the potential for the recovery of articular cartilage with regard to different tissue conditions after they had undergone friction tests with different pressures [12]. After the friction tests, they observed a change in the cartilage thickness of the pin of up to 70%. They also noticed tracks in the cartilage plate with a depth of up to approx. 0.3 mm [12]. The assessed cartilage was artificially GAG deficient. In the present study, we observed HL in the range from 0.22 mm (E48w) to 0.33 mm (MC24w). Even considering the fact that we measured the HL under a pressure of 0.75 MPa, our results are still comparable with the results of Katta et al. [12]. 

The “tribological rehydration” phenomenon was discovered by Moore and Burris [35,60] when they assessed data from measurements of the compression of cartilage under a normal load of 5 N with an LVDT. They used flat and convex-shaped cartilage samples from mature bovine stifle joints in their in situ tribometer. 

Creep could be a further reason for the HL of the AC and regenerated AC tissue that occurs under compression. Gao et al. [61] investigated the responses of three different zones of AC from the knee joints of an about 8-month-old pig in an unconfined compression setup [61]. They found that the Young’s Modulus increased along the depth of the AC and the Young’s Modulus of the layers of the AC also increased, depending on the stress rate. Thus, the deformation of the tissue should depend on the mechanical and structural characteristics of the composition of the assessed tissue and the type of stress. In an investigation of the frictional response under creep conditions, Basalo et al. [55] found a constant creep strain of 0.55 for bovine articular cartilage, both in the control group and in the group that was treated with chondroitinase ABC. However, in their experiments, they had removed the deep zone of the tissue. 

Diermeier et al. [62] investigated the effect of a focal metallic implant on the opposite cartilage in an abrasion machine. They focused on the damages of the zones of the articular cartilage of knees from young pigs. They found damages of the tangential and of the radial zone after 6 h [62].

In another study, we compared the HL of degenerated cartilage with the HL of healthy cartilage of pigs from the slaughterhouse [19]. In both groups, we observed that the main HL occurred within the first 100 to 300 cycles in a comparable testing setup. The curve progressions in the present study (Figure 3a) are comparable with the results published by [19]. Thus, the determination of the HL seems to be a useful criterion for the determination of the mechanical properties of AC, even if the mechanical parameters for that behavior are not yet clear [19].

#### 4.7.3. HL Useful for Differentiation

The determination of the HL under tribological exposure was possible because we used a unique tribometer equipped with a vertical distance sensor for the determination of the slope angles of an uneven surface [2]. The data provided by the vertical sensor made it possible to identify the HL during each cycle (Figure 3a and Figure S3a). It is an advantage of the tribological examination that the data of the HLs are directly and almost automatically recorded by the tribometer system. However, it can be seen as a disadvantage that we cannot differentiate whether the HL is mainly caused by the material properties of the (regenerated) cartilage tissue on top of the pin or whether it is caused by the material properties of the cartilage layer of the plate. In this context, one has to keep in mind that the cartilage layer of the tibia plateaus could possibly possess worse mechanical properties than that of the femoral site [63,64]. Thus, the comparison of the results of the different treatments can be impeded because the tissue in question was located on top of the pin. Thus, future studies should implement separate measuring strategies for the analysis of the HL of the cartilage layer of the pin and that of the plate. Katta et al. [12] did so in their study dealing with the GAG-deficient cartilage in friction tests [12].

However, the determination of the HL after tribological exposure could help to determine the stability of regenerated cartilage against shear stress after a variety of treatments. A significant difference between degenerated and healthy cartilage regarding the resilience of AC, expressed as HL, has been reported in a recent study [19].

In the future, this could be an easier testing protocol than the determination of several mechanical properties, in particular, those such as interstitial fluid pressurization, Young’s modulus, creep, etc. [37,61]. By comparing regenerated cartilage with natural cartilage, we were able to show significant differences in the HL of cartilage layers after tribological exposure between groups that had undergone different operational procedures. We believe that we are one of the first to perform such a comparison with regenerated cartilage tissue (Figure 3b). 

#### 4.7.4. HL Regarding Internal and External Controls

The comparison of the HL of the regenerated cartilage and that of the internal controls of the operated groups revealed only one significant difference within the M24w group. There was an increase of 45% in the HL. The value of the HL of this group (M24w) is double that of the natural cartilage in the defect area. 

Thus, the comparison of the HL with natural cartilage as a control tissue promises a better differentiation between the groups.

### 4.8. Effect Sizes (ES)

In the presented study, we followed the idea to calculate the ES for the comparisons between the NAT group and the operated groups regarding both the CoF and the HL, as recently recommended in our article [22]. The ES can help to better visualize the differences between the control group and the treated groups because the differences of the mean values are adjusted by the pooled standard deviations [26]. As shown in Figure 4, the ESs of the CoF showed small deviations for all assessed groups, although they are high, with 1.3 in the mean. This means that we were able to detect the inferiority of the CoFs of the regenerated AC in comparison with natural AC tissue. 

Looking at the ESs of the CoF, we noticed a large effect size similar to that of the HL, with 1.3 and 1.39 in the mean, respectively. However, for the latter, some variations were noticed in the groups where the defects had been treated with implants (M24w, M48w, MC24w, and MC48w). Larger ESs were noted after a healing period of 24 weeks than after a healing period of 48 weeks (Figure 4). Thus, we assume that the healing effects of the assessed tissues are reflected in those data.

We described the usefulness of the determination of the ES to justify the sample size calculation when several types of analyses are included in a study, as was the case in the presented study [22]. Looking at Figure 5 of that study [22], we can guess the power of the results in the present study in the range from approx. 60% to approx. 80% for the CoF. In some cases, the range may be higher, as is the case for the HL, with 2.04 in the M24w group where we were able to examine all 10 samples as had been determined by the sample size calculated prior to the study as reported [22]. 

### 4.9. Limitations and Advantages of the Study

We did not provide any data that could help understand what type of friction occurred in the different types of regenerated cartilage and at which point in time during the examinations. Thus, we were not able to analyze if and when the friction regime changed from fluid film lubrication to boundary lubrication. It could be a topic of future studies to analyze the contents of water or GAGs in the assessed tissue or in the lubricant before and after the tribological exposure. 

We were not able to quantify the different contributing mechanical properties that affected the HL in particular, like creep, fluid pressurization, aggregate modulus, or the volume loss of the tissue through wear. Neither did we analyze where the loss occurred, on the pin or on the plate, even though we observed and recorded that shreds of tissue were torn off from the layers of the pins, but we had no clear notion of how to quantify (Figure 1). 

It is not clear to what extent CoF and HL measurements could be affected by a misalignment between the surface area of the pin and the uneven surface of the plate and if a better alignment could be achieved after some cycles. Further investigations should study the contact area between the pin and plate in particular. Nevertheless, the data from the natural cartilage showed quite homogenous curve progressions in the groups regarding the CoF and HL, respectively (Figures S2a,b and S3a,b). Furthermore, in contrast to other studies dealing with regenerates [11], we identified significant statistical differences between the regenerated AC tissue and natural AC in terms of the assessed CoF, using the presented protocols. 

The determination of the differences in CoF and HL was made possible by using our custom-made tribometer [2]. Future studies could address the question of whether or not the CoF and the HL are independent variables, and if so, under which conditions. 

## 5. Conclusions

The constantly low value of the CoF of articular cartilage was confirmed by the results and corresponds to reports in literature when cartilage was moved against cartilage. The measurement of the height loss (HL) of articular pair specimens was introduced as a potential additional criterion to appraise the mechanical stability of natural and regenerated articular cartilage under tribological exposure. 

The mechanical quality of the regenerated AC tissue showed inferiority with regard to the CoF and HL in comparison with natural AC. 

Natural AC from untreated joints seems to be the better control tissue as more significances were detected than by the comparison with cartilage from the joints where the regenerates were created.

## Figures and Tables

**Table 1:** The labeling of the groups and the different treatments of the defects. Each group consisted of 10 animals. The NAT group served as the external natural control because the stifle joints of the animals were not operated. The Col I matrices that the animals of the MC24w and the MC48 groups received were laden with autologous cells isolated from the removed cartilage tissue of the defects.

Group	Filling of Defects	Observation Period	Number of Animals (*n*)
NAT	no defects set; external control	24 weeks	10
E24w	no filling, empty defects	24 weeks	10
E48w	no filling, empty defects	48 weeks	10
M24w	matrices without cells	24 weeks	10
M48w	matrices without cells	48 weeks	10
MC24w	matrices laden with autologous cells	24 weeks	10
MC48w	matrices laden with autologous cells	48 weeks	10

## Supplementary Materials

The following supporting information can be downloaded at: https://www.mdpi.com/article/10.3390/biomedicines10112685/s1, Figure S1: Sketch of the facets of the trochleae and the distribution of the defect areas (O) where the 6 mm defects were set (according to Schwarz et al., 2019, [22]). The areas next to the defect area (grey) were referred to as non-defect areas. In the defect areas, we harvested the regenerated cartilage tissue, and in the non-defect areas, we harvested the articular cartilage, as osteochondral pins with a diameter of 5 mm as “internal” controls [27]. Figure S2: The CoFs from the non-defect areas. (a) The figure shows the trends of the CoF during the examination duration of approximately 1 h (554 cycles). For the sample size per group, see figure (b). (b) The CoFs showed no significant differences (n.s.) between the NAT group and each individually operated group. Figure S3: The HLs from the non-defect areas. (a) The figure shows the trends of the HL during the examination duration of approximately 1 h (554 cycles). For the sample size, see figure (b). (b) The HLs showed no significant differences (n.s.) between the NAT group and each individually operated group.
