# Supplementary material for: Coefficient of Friction and Height Loss: Two Criteria Used to Determine the Mechanical Property and Stability of Regenerated Versus Natural Articular Cartilage
Source: Biomedicines. 2022 Oct 24;10(11):2685. doi: 10.3390/biomedicines10112685 (PMC9687335; doi:10.3390/biomedicines10112685)
Supplement: Supplementary file 1 [file biomedicines-10-02685-s001.zip › biomedicines-1916887-supplementary.pdf]

Supplementary figures

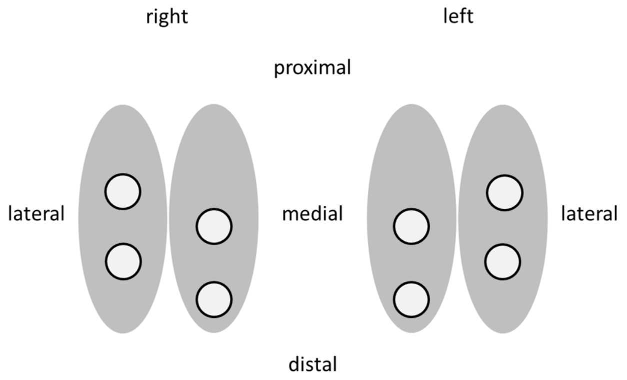

Figure S1. Sketch of the facets of the trochleae and the distribution of the defect areas (O) where the 6 mm defects were set (according to Schwarz et al. 2019, (22)). The areas next to the defect area (grey) were referred to as non-defect areas. In the defect areas, we harvested the regenerated cartilage tissue, and in the non-defect areas, we harvested the articular cartilage, as osteochondral pins with a diameter of 5 mm as "internal" controls (27).

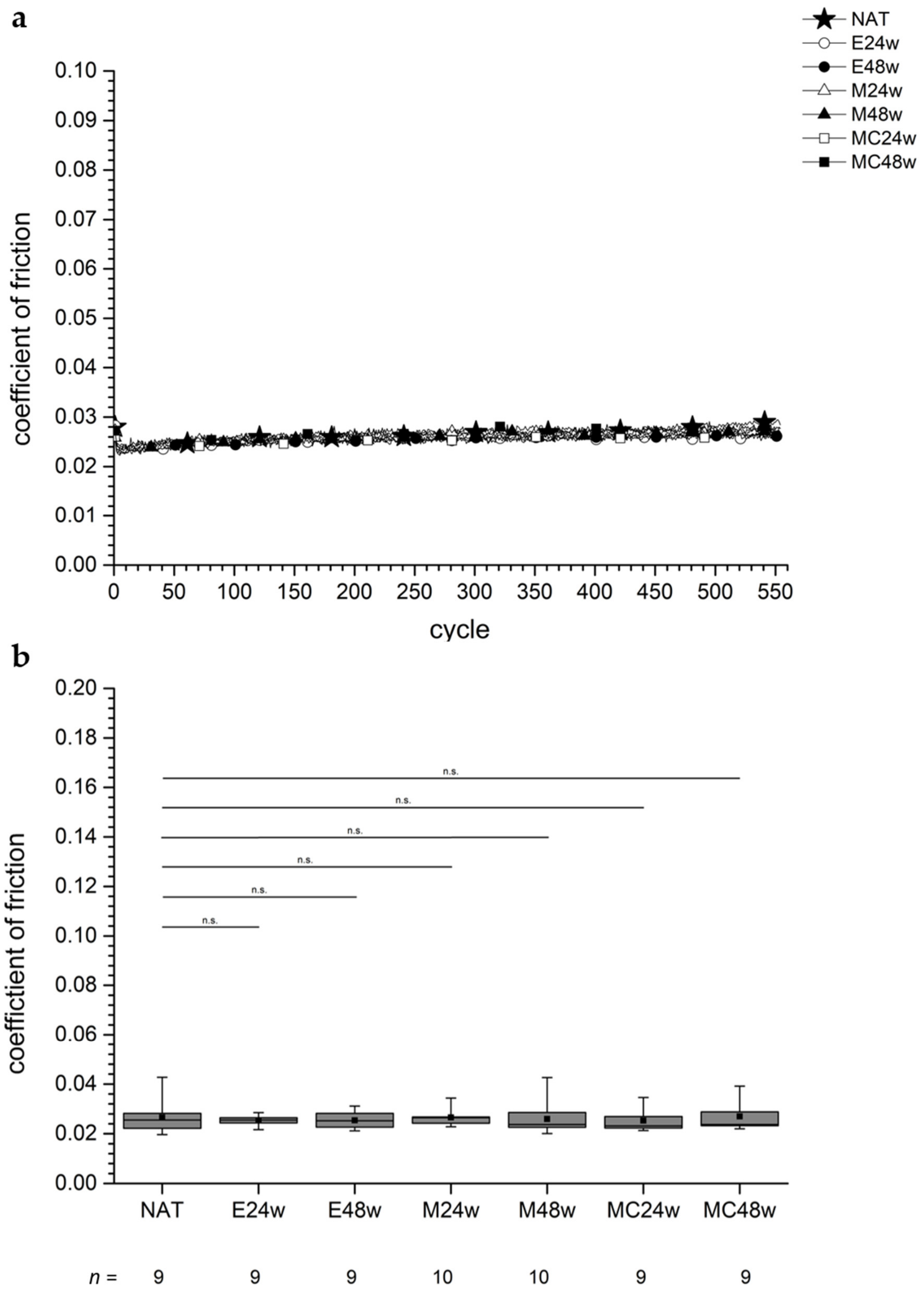

Figure S2. The CoFs from the non-defect areas. (**a**) The figure shows the trends of the CoF during the examination duration of approximately 1 h (554 cycles). For the sample size per group, see figure (**b**). (**b**) The CoFs showed no significant differences (n.s.) between the NAT group and each individually operated group.

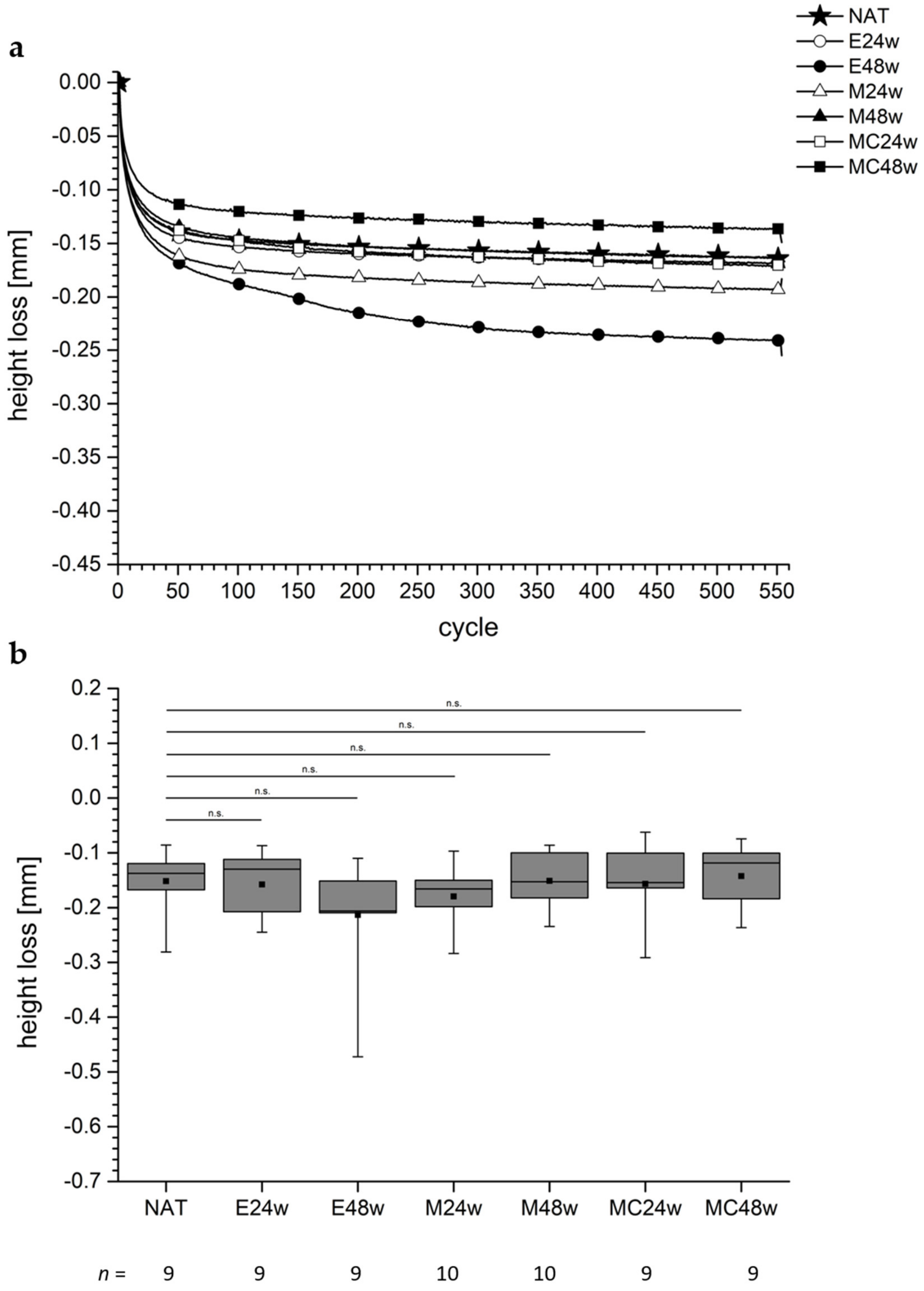

Figure S3. The HLs from the non-defect areas. (**a**) The figure shows the trends of the HL during the examination duration of approximately 1 h (554 cycles). For the sample size, see figure (**b**). (**b**) The HLs showed no significant differences (n.s.) between the NAT group and each individually operated group.
